# Progress in Surgery

**Published:** 1899-01-28

**Authors:** 


### PROGRESS IN SURGERY.

### GYNÆCOLOGY AND OBSTETRICS.

(*Continued from page* 181.)

**Conservative Surgery of the Ovary.**—Christopher Martin[17] advocates conservative treatment of the ovaries whenever possible, and points out how small a fragment of ovarian tissue is necessary to preserve the full influence of the gland on the body. He says that in cases of ordinary cystoma of one ovary, the other being healthy, it is unjustifiable to remove both. If, however, the case be one of sarcoma of the ovary, even if the other ovary appear normal, both should be removed, as sarcoma is so extremely apt to affect one ovary and then the other. In cases of inflammatory disease of the uterine appendages (salpingitis, pyosalpinx, ovarian abscess, &c.), if the disease be confined to the organs on one side only it is unnecessary to remove them on both sides; in cases of double pyosalpinx we should endeavour to leave behind one or both ovaries unless they are obviously diseased, and he quotes cases where he has been able to leave some part of the ovary after operating for fibroma of ovary, dermoid tumour, and cystic disease. In cases where the cystic disease affected only one pole of the ovary he has removed a wedge which included all the diseased tissue and then sewn up the wedge-shaped gap by a fine continuous silk suture. In cases of chronic ovaritis associated with severe dysmenorrhœas, menorrhagia, ovarian neuralgia, hysteria, &c., instead of removal of ovaries he recommends the conservative operation of ignipuncture. The peritoneum is opened through the vagina. Any adhesions binding down the ovary are separated with the finger, the ovary punctured with a fine point of Pacquelin's cautery, heated red-hot, wherever there is an indication of a cyst in the cortex or an unusually thick portion of the capsule of the ovary. The red-hot point is inserted in this way in from six to a dozen punctures each about one-twelfth of an inch in diameter; the appendage having been replaced, the vaginal wound is closed with catgut. Martin has performed this operation for cystic ovaries fourteen times, in seven of which a cure has resulted. In cases of parovarian cyst and cyst of the broad ligament the cyst should be shelled out and the ovary preserved. In cases of vaginal or abdominal hysterectomy one or both ovaries should be left behind. Dr. Palmer Dudley,[18] of New York, has performed one hundred and three conservative operations on the uterine appendages without a death and with inflammation in only one case.

**The Use and Abuse of Midwifery Forceps.**—Dr. Milne Murray,[19] at the Edinburgh meeting of the British Medical Association, says that the value of the forceps as a surgical instrument consists in the power it gives us on certain occasions of shortening the duration o labour, of diminishing the dangers of the mother, and greatly increasing the safety of the child. Discussing when the forceps should be used he lays down as a general principle that a direct indication for the use of the forceps arises whenever—and only whenever—we are assured that the danger of interference has become less than that of leaving the patient alone. The reason that the question of the proper use of the forceps is often so difficult to answer is that much more than in most surgical operations have we to consider in each not one but many circumstances. Milne Murray says that (*a*) forceps are often used at the wrong times; (*b*) they are sometimes not used at the right times; (*c*) they are often badly used at both times. The dangers of the forceps operation apart from the risk of septic infection (which, of course, is common to most operations) may be summarised thus: (1) The mother's parts may be bruised, lacerated, or otherwise injured by mechanical violence. (2) The too sudden emptying of the uterus may be followed by imperfect retraction, and consequently dangerous hæmorrhage. (3) The fœtal head may be unduly compressed, lacerated, or otherwise damaged. Dr. Murray lays stress on the great danger of using the forceps in undilated os, especially in spasmodic rigidity; also in secondary inertia when post partum hæmorrhage is so likely to be a result. In answer to the question, When should the forceps be applied? he says: "When the passages are in a fit state, and nature fails to advance the head," and he protests against the idea that the time element alone is a proper basis for such interference. In unreduced occipito-posterior positions he believes that after the os is sufficiently dilated the sooner the forceps are applied the better. As to the pattern of forceps, he strongly recommends the axis traction, whether the head be at the brim, in the cavity, or at the outlet, and holds that deep anæsthesia, combined with the skilful use of axis traction forceps, is the best treatment for rigid perineum. He condemns the teaching and practice of most of the British schools to apply the forceps as far as possible in relation to the pelvic transverse without reference to the position of the head, and thinks the practice of the French schools is much sounder—that the forceps should be applied to the bi-parietal diameter of the child's head wherever situated, and then as the head descends rotation will bring the blades into the transverse diameter, and the head, forceps, and pelvis will then present proper relations at the outlet. He believes that extensive damage to the lower part of the vaginal canal, especially internal laceration of the perineum, is often caused by oblique position of the head or of the forceps at the outlet.

**Treatment of Rupture of the Uterus.**—Mayo Robson,[20] writing on the treatment of rupture of the uterus, says that when rupture occurs before delivery and the child escapes into the abdominal cavity nothing but immediate abdominal section can offer any chance of saving the patient, and in such cases interference must be prompt both on account of the hæmorrhage and shock; but in those cases of rupture where the laceration is in the lower segment of the womb, and when the rent occurs during delivery there is, as a rule, more time for consideration, as the shock, though great, may not be immediate and the hæmorrhage may not be so violent as to produce immediate collapse. A simple vaginal examination may not discover the rupture, as the lower segment of the cervix is not necessarily torn, but if the finger be passed within the os the rent in the uterus will be felt and the symptoms explained. Probably the best treatment in these cases is to pack the uterus with iodoform gauze. Mayo Robson gives the details of a case in which he adopted this treatment with complete success; no further bleeding occurred and no peritonitis ensued. In the clinical report of the Rotunda Hospital[21] for this year a case of rupture of the uterus is recorded which was treated in this way. The rent in the uterine wall was about three inches in length, situated in the lower segment to the right and posterior; a thick plug of iodoform gauze was passed through the rent into the peritoneal cavity for the purpose both of drainage and to keep the intestines out of the wound, the lower end of the gauze being in the vagina. The patient made an uninterrupted recovery.

**Aseptic Midwifery.**—Dr. Jardine[22] read a paper on this subject at the Edinburgh meeting of the British Medical Association this year. He points out that parturition may be said to be a physiological act, but among highly civilised women it is dangerously near a pathological one, and very little may turn the scale. In the vast majority of cases at the onset of labour the genital tract is perfectly aseptic: by this we do not mean that it is free from organisms; on the contrary, it is swarming with them, at least the vagina and lower part of the cervical canal, but these organisms are not only non-pathogenic but they are actually protective, preventing the pathogenic ones gaining a foothold by rendering the vaginal secretion acid. The vagina has within it a self-cleansing power. The cervical canal has a plug of mucus—the operculum—which shuts off as it were the uterine contents from the vagina. The different layers of this plug have been examined bacteriologically, and the upper or uterine layer has been found to be perfectly free from organisms. We therefore start with an aseptic uterus and genital tract; the discharges which come from within, the liquor amnii and blood will not in any way interfere with that condition, but tend rather to assist it by flushing out to a certain extent anything which may get into the lower part of the genital tract. Our whole aim is to conduct the labour so that at the end the tract will be as aseptic as when we began. The treatment during the puerperium must also aim at the same thing. He points out that the genital tract may be infected first and most frequently from the examining finger; secondly, from the use of septic instruments; thirdly, from aseptic fingers or instruments carrying in infection from the external parts of the patient or the surrounding clothing. The attendant's hands must be surgically clean, the external genitals must be thoroughly cleansed, and vaginal examinations must be as few as possible. As to douching in an ordinary case, it is unnecessary, and in fact as likely to do harm as good by removing the normal secretion from the vagina. He considers it necessary under the following conditions: (1) if there is any purulent or putrid discharge from the vagina such as from gonorrhœa or cancer of the cervix; (2) if any operation is to be performed when the hand or any instruments have to be introduced into the uterus. Post partum douching is not necessary in ordinary cases, and should not be done as a routine. Under the following conditions one should douche immediately after labour: (1) in post partum hæmorrhage, then it should be given very hot; (2) if there has been any purulent discharge previous to labour; (3) if the fœtus has been putrid: (4) if the hands or instruments have been introduced into the uterus; (5) if the parts have been lacerated to any extent and if the labour has been a very prolonged one. During the puerperium douching is quite unnecessary unless the lochia become putrid and the temperature rises. The second stage of labour must not be allowed to drag on indefinitely. Dr. Jardine thinks two hours for the second stage in a multipara and three hours for a primipara is quite long enough; if no advance is then being made it is time to interfere. The third stage must be conducted very carefully to make sure that nothing is left behind in the uterus. As soon as the third stage is complete and the uterus remains firmly contracted all soiled things must be removed from the bed and the patient thoroughly washed with an antiseptic. During the puerperium no soiled things should be allowed to remain under the patient or in the room; the napkins must be changed when soiled, and the soiled ones burnt. The external genitals washed frequently with a warm antiseptic. If a douch should become necessary perchloride of mercury 1-2,000, followed by sterilized water, is the best.

**The Vomiting of Pregnancy.**—Bacon[23] says that vomitus gravidarum may be defined as vomiting during pregnancy due to a variety of immediate causes acting upon the abnormally irritable nervous system of the pregnant woman. There are various possible ways of explaining vomitus gravidarum: (*a*) Direct vomiting may be produced by an abnormal condition of the vomiting centre due either to the irritating effects of chemical substances, toxins, &c., circulating in the blood or to nutritional changes caused by variations in the blood pressure in the medulla or to other circulatory changes; (*b*) reflex vomiting may be produced by sufficiently powerful impulses sent from the genital tract, causing an irritation of the vomiting centre; (*c*) vomiting may be produced by a combination of influences affecting the vomiting centre both directly and reflexly; (*d*) still another possible cause of vomitus gravidarum is the psychopathic factor like that which exists in the vomiting of hysteria. The increased irritability of the nervous system during pregnancy is a well-known and established clinica fact, as is shown by the changed mental condition, the disturbance in taste, sight, and hearing, and by the fact recently observed by Neumann of increase in the patella and other reflexes. Bacon sums up the suggestions regarding treatment as follows: (1) The abnormal irritability of the nervous system, including the vomiting centre, is to be allayed by keeping the patient in the horizontal position and by attention to the bowels, skin, and kidney; (2) the hysterical condition which is so commonly found present should be controlled; (3) all sources of peripheral irritation should be discovered and treated; (4) in extreme case subcutaneous saline injections serve the threefold purpose of (*a*) dilating the blood vessels and increasing the vascular tension, (*b*) eliminating toxins through renal and intestinal emunctories, (*c*) furnishing two most important kinds of food; (5) induction of abortion is never indicated. At a stage when it is safe and efficient it is not necessary, and in extreme cases it adds greatly to the danger.

17 Brit. Med. Jour., Sept. 17, 1898. 18 Med. Rec., June, 1898. 19 Brit. Med. Jour., Aug. 20, 1898. 20 Practitioner, July, 1898. 21 Dublin Jour. of Med. Sci., June, 1898. 22 Brit. Med. Jour., Sept. 17, 1898. 23 Amer. Jour. of Obstetrical Society, June, 1898.

